# Neurocognitive effects of androgen deprivation therapy and new hormonal agents in a sample of patients with metastatic prostate cancer

**DOI:** 10.1007/s11255-023-03712-z

**Published:** 2023-07-19

**Authors:** Andreas Ihrig, Pascal Marino Pernt, Stefanie Zschäbitz, Johannes Huber, Hans-Christoph Friederich, Till J. Bugaj, Imad Maatouk

**Affiliations:** 1 Division of Psycho-Oncology, Department of General Internal Medicine and Psychosomatics, University Hospital Heidelberg, INF 410, 69120 Heidelberg, Germany; 2 Department of Medical Oncology, National Centre for Tumor Diseases (NCT), University Hospital Heidelberg, Heidelberg, Germany; 3 Department of Urology, Philipps-University Marburg, Marburg, Germany; 4 Section of Psychosomatic Medicine, Psychotherapy and Psycho-Oncology, Department of Internal Medicine II, Julius-Maximilian University Würzburg, Würzburg, Germany

**Keywords:** Metastatic prostate cancer, Hormonal agents, Androgen deprivation therapy, Neurocognitive effects

## Abstract

**Introduction:**

Although the growing treatment landscape for metastatic prostate cancer (mPC) has revealed new opportunities, it has also provided challenges, such as undesirable side effects. The aim of the present study was to provide further data on domain-specific cognitive impairments in mPC patients with androgen deprivation therapy (ADT) and new hormonal agents.

**Methods:**

Fifty-eight patients (71 ± 8 years) with mPC were investigated using a cross-sectional design. All patients had received some form of ADT (93% had received luteinizing hormone-releasing hormone (LHRH) analogs/antagonists), 66% had received chemotherapy, and 84% had received anti-resorptive therapy. We evaluated learning and memory, processing speed, and executive functions, as recommended by the International Cognition and Cancer Task Force, to determine neurocognitive deficits.

**Results:**

Patients treated with ADT scored significantly lower on all neurocognitive tests and showed significantly more neurocognitive deficits (38–62%) than age-adjusted reference samples (16%, *p* < 0.05). Cognitive deficits were mild in most cases and predominantly affected visuomotor processing speed (48%). Moderate and severe deficits were found in 11% and 5% of patients, respectively, with word fluency as the predominant deficit (23%). No associations were found between the type or duration of treatment and the severity of cognitive deficits.

**Conclusions:**

Treatment of mPC with ADT is correlated with neurocognitive deficits in several cognitive domains. Language skills and processing speed were most frequently impaired. However, a consistent pattern of cognitive impairment was not identified. Neurocognitive deficits should be considered in phase III and IV trials.

**Trial registration:**

The study was registered in the German Clinical Trials Registry (DRKS00017727).

## Introduction

Androgen deprivation therapy (ADT) is one of the most effective treatments for advanced prostate cancer (PC) [1]. In metastatic disease and nonmetastatic castration-resistant disease, combination therapies consisting of luteinizing hormone-releasing hormone (LHRH) agonists or antagonists and new hormonal agents such as apalutamide, enzalutamide, abiraterone acetate and docetaxel are used [2].

The goal of all treatments is to slow the progression of disease, prolong overall survival, relieve cancer-specific symptoms, and maintain or improve quality of life. Nevertheless, chemotherapies also have undesirable side effects, such as the so-called chemobrain [3]. This describes a constellation of cognitive deficits that patients experience during or after chemotherapies. In addition, PC patients (between 10% and 69%) experience treatment-related cognitive deficits [4]. Several studies have implicated treatment-specific relationships between cognitive deficits and systemic PC treatment [4–6]. These cognitive problems may persist for years after treatment [7].

However, studies on treatment-specific cognitive effects have yielded inconsistent results [5]. Thus, a larger empirical base is needed, especially to determine which neurocognitive domains are affected, as emphasized in several reviews [4–6].

The International Cognition and Cancer Task Force (ICCTF) formulated guidelines to increase the comparability of research findings on neurocognitive deficits in cancer patients and to better structure research in this area [8]. Domains that are frequently associated with cancer treatment-related neurocognitive deficits are language skills, learning and memory, and executive functions, including processing speed [8].

The aim of the present study was to provide further data on domain-specific cognitive impairments in metastatic PC (mPC) patients.

## Methods

The study was conducted at the National Center for Tumor Diseases (NCT) in Heidelberg. Approval for the study was obtained from the Ethics Committee of the Medical Faculty of the University of Heidelberg (S-178/2019). The study was registered in the German Clinical Trials Registry (DRKS00017727).

The inclusion criteria were as follows: diagnosis of mPC, age above 50 years and having received one or more ADT courses. The exclusion criteria were as follows: poor German language skills (i.e., unable to answer questionnaires), visual or hearing impairment, known brain metastases, or a prior diagnosis of severe neurological or psychiatric disease.

Patients with mPC undergoing ADT were eligible if they received treatment at the NCT during the data collection period (from May to August 2019). Patients were recruited on site. After providing informed consent, they completed a neurocognitive test and answered an anamnestic questionnaire. Medical characteristics were extracted from each patient's medical record.

We classified the treatments into five categories:

1. Drug (LHRH analogs/antagonists) or surgical (bilateral orchiectomy) castration (ADT)
2. ADT (bicalutamide)
3. ADT plus CYP17 inhibition (abiraterone acetate)
4. ADT plus androgen receptor inhibition (enzalutamide)
5. ADT plus cytostatic drugs (docetaxel, cabazitaxel, etc.)

The Vienna Test System (WTS) of Schuhfried GmbH was used to assemble a neuropsychological test battery, since it contains reference values for men of higher age [9, 10]. This battery included the following tests: the Trail Making Test (TMT), which measures attention, processing speed, and mental flexibility; the Vienna Word Fluency Test (WIWO), which measures lexical word fluency; the Auditory Word List Learning Test (AWLT), which assesses deficits in long-term verbal memory and verbal learning ability; and the N-Back verbal (NBV) working memory task, which measures verbal working memory. This compilation corresponds to the ICCTF test recommendations [8].

For each neuropsychological test, normal values measured in the normal population are available for men from different age groups. Each test result was classified in comparison with the results of the matching age group. Classifications range from normal (< 1.0 standard deviation (SD) below the age and gender-adjusted reference value) to slight (1.0–1.5 SD), moderate (1.5–2.0 SD) and severe deficits (> 2.0 SD). According to the ICCTF, a cross-test neurocognitive deficit is defined as at least one subtest score 2 SD below the mean or at least two subtest scores 1.5 SD below the mean [8]. To quantify the test-wide deficits, an average Global Deficit Score (GDS) was calculated [8, 11].

### Statistical analysis

The results are presented as the frequency (percentage) or as the mean ± standard deviation. For the comparison of observed vs. expected frequencies, z tests were used [12]. One-sample *t* tests were used to test deviations of the drawn sample from age-, education-, and sex-matched reference data on neurocognitive domains. To determine the direction and magnitude of the correlations between the medical treatment variables and the GDS, multiple regressions were used.

## Results

Out of 72 patients addressed, 58 (81%) agreed to participate. The demographic and clinical characteristics of the sample are presented in Table 1.

**Table 1:** Demographic and clinical characteristics of the sample (*n* = 58)

Demographic characteristics	*n* (%)	Median	Range (min–max)
Age (years)	–	72.0	50.0–82.8
In a relationship (*n* = 57)	50 (87.7%)	–	–
Has children	49 (84.5%)	–	–
Education: A-level	29 (50%)		
Employed (vs. retired; *n* = *56*)	13 (23.2%)	–	–
Diagnosis-specific data			
Time since first diagnosis (years; *n* = 57)	–	4.3	0.1–20.7
Initial prostate specific antigen (PSA) value (µg/l; *n* = 43)	–	42.5	2.9–1892
Gleason score (*n* = 52)			
6	3 (5.8%)	–	–
7	10 (19.2%)	–	–
8–10	39 (75%)	–	–
Metastases		–	–
Bone	47 (82.5%)	–	–
Lymph node	39 (68.4%)	–	–
Other site	13 (22.8%)	–	–
Castration-resistant metastatic prostate cancer (mCRCP)	42 (72.4%)	–	–
Charlson Comorbidity Index			
0	34 (59.6%)	–	–
1	14 (24.6%)	–	–
≥ 2	9 (15.8%)	–	–

The mean age of the participants was 70.9 ± 8.4 years. Most patients were retired, had a partner and had children. The median time since diagnosis was 4.3 years. Most patients had metastases, a castration-resistant cancer and a Charlson Comorbidity Index of 0.

During treatment, 50% of the patients received radical prostatectomy, 49% received external beam radiotherapy, and 12% received brachytherapy. All patients had received some form of androgen deprivation therapy (93% had received LHRH analogs/antagonists), 66% had received some form of chemotherapy (docetaxel, cabazitaxel, carboplatin/etoposide) and 84% had received anti-resorptive therapy (denosumab/zoledronic acid). Details of treatment-specific characteristics are presented in Table 2.

**Table 2:** Overview of treatment-specific characteristics

Treatment	At test	During treatment:	Cumulative treatment duration (months)/number of cycles		
	*n* (%)	*n* (%)	Md (treated)	Range (treated)	Md (total)
Local treatment					
Radical prostatectomy		29 (50%)	–	–	–
Brachytherapy	0 (0%)	7 (12%)	–	–	–
External beam radiotherapy (*n* = *57*)	0 (0%)	28 (49%)	–	–	–
Androgen deprivation therapy	58 (100%)	58 (100%)	–	1.3–205.4	38.3 months
LHRH analog/antagonist (*n* = *57*)	51 (89%)	54 (93%)	42.4 months	0.3–205.4	38.3 months
Bicalutamide	10 (17%)	37 (64%)	8 months	0.5–151.5	1.9 months
Enzalutamide	14 (24%)	21 (36.2%)	5 months	1.0–56.0	0 months
Abiraterone acetate	10 (17%)	27 (47%)	10 months	1.0–37.0	0 months
Cytostatics	16 (28%)	38 (66%)	6 cycles	1–16	4 cycles
Docetaxel	14 (24%)	37 (64%)	6 cycles	1–16	3.5 cycles
Cabazitaxel	1 (2%)	5 (9%)	4 cycles	2–6	0 cycles
Carboplatin/etoposide	1 (2%)	3 (5%)	3 cycles	2–6	0 cycles
Other cancer-specific therapies					
Denosumab/zoledronic acid (*n* = *57*)	47 (82%)	47 (82%)	–	–	–
Prostate-specific membrane antigen (PSMA) ligand therapy	1 (2%)	1 (2%)	–	–	–
Checkpoint inhibitor	5 (9%)	6 (10%)	–	–	–
PARP inhibitor	3 (5%)	3 (5%)	–	–	–
Any pain medication	6 (10%)	–	–	–	–
Antidepressant	2 (3%)	–	–	–	–

We summarized the results of neurocognitive tests in Table 3. The t test results revealed significantly lower test scores in cognitive domains compared to the reference data except for one subtest (learning total). In all tests, the percentage of patients with at least one subtest indicating mild deficits was significantly higher than in the age-adjusted general population. In the WIWO, 43% of the participants scored significantly lower than the reference group, and 11% of them exhibited severe deficits. In addition, in the AWLT, significantly more patients exhibited deficient results (38%), with 17% of them displaying moderate or severe deficits. In the TMT and NBV test, 60% and 62% of participants, respectively, exhibited significant deficits, with an increase in frequency mainly of the slight-deficit group (48%/38%).

**Table 3:** Neurocognitive test results

Test	*t* test results				Frequency of deficits (*n*, %)			Sum > 1.0 SD
	M	SD	*t*	*p* value	Slight 1.0–1.5 SD	Moderate 1.5–2.0 SD	Severe < 2.0 SD	
WIWO (word fluency), *n* = 56	41.7	8.3	− 6.32	< 0.001	11 (20%)	7 (13%)	6 (11%)	24 (43%)*
AWLT (memory, learning), *n* = 55								
Learning total	50.5	9.7	0.38	*n.s*	12 (22%)	8 (15%)	1 (2%)	21 (38%)*
Short-term delayed retrieval	45.9	10.0	3.12	< 0.01				
TMT (attention, speed), *n* = 52								
Processing time (TMT-A)	40.9	4.7	6.93	< 0.001	25 (48%)	5 (10%)	1 (2%)	31 (60%)*
Processing time (TMT-B)	47.2	6.8	2.13	< 0.05				
NBV test (working memory), *n* = 50								
Correct	44.2	6.5	4.10	< 0.001	22 (38%)	4 (8%)	2 (4%)	28 (62%)*
Incorrect	45.1	7.1	3.46	< 0.01				

*p < 0.05

A total of 51 patients completed three or four cognitive tests. Of these participants, 47 (92%) had at least one neurocognitive test indicating a slight deficit. This percentage was significantly higher than expected according to the reference sample (*p* > 0.001). A cross-test neurocognitive deficit, defined by having at least one subtest score 2 SD below the mean or at least two subtest scores 1.5 SD below the mean, was observed in 13 (26%) patients. According to the ICCTF definition, on the three tests (WIWO, AWLT, TMT), 10 (17%) of all patients showed a cross-test neurocognitive deficit. This percentage is not significantly higher than expected according to the reference sample.

The T-transformed GDS was 44.9 ± 4.3 (36.1–54.9). Seventeen (30%) patients had GDSs above the cutoff point of ≥ 0.50 [11]. Two multiple regressions were used to examine the correlations of GDSs with the type and duration of medical treatment (results shown in the Appendix). The type of treatment showed no significant correlation with cognitive performance. The duration of treatment with abiraterone acetate had a significant positive correlation with the GDS, whereas longer treatment with drug/surgical castration predicted significantly lower GDS. The overall predictive model had an R^2^ value of 0.17.

## Discussion

Patients with mPC scored lower in all neurocognitive tests and had more frequent neurocognitive deficits than reference samples. The results suggest that treatment for mPC is correlated with mild neurocognitive deficits.

The frequencies of mild cognitive deficits were significantly above the expected frequencies. Almost all patients (92%) showed at least a slight cognitive deficit in at least one test. In contrast, moderate and severe deficits were relatively rare in our sample. These frequencies are also greater than those described by the ICCTF. Notably, our study included not only the three recommended tests of learning and memory, processing speed, and executive function but also the NBV test of verbal working memory. Excluding the NBV test results reduced the frequency of patients with a cross-test neurocognitive deficit from 13 to 10, a frequency not significantly higher than that expected in a healthy reference group (7).

Compared to the reference sample, patients treated for mPC had the most significant deficits in word fluency (in the WIWO) and visuomotor processing speed (in the TMT-A). Word fluency is also the only domain in which significantly more severe neurocognitive deficits were observed. Regarding verbal long-term memory (measured with the AWLT short-term delayed recall) and executive function (measured with the TMT-B and NBV test), significantly lower performance and increased mild cognitive impairments were observed in patients treated for mPC. Verbal learning ability, assessed with immediate recall/learning sum in the AWLT, is the only subtest in which patients did not significantly differ from the reference sample. These results are consistent with a recent review of men with advanced prostate cancer and suggest that treatment with ADT may increase the risk of cognitive impairment [6]. Overall, the available data are unclear. In two long-term studies, no consistent trend in terms of the affected neurocognitive domain was found [13, 14]. In addition, two reviews did not obtain consistent results concerning ADT-induced cognitive impairment [15, 16].

In our sample, we found no evidence that a single type of treatment predicted cognitive performance. Treatment duration had a few partial correlations that supported our hypothesis. However, these analyses are only exploratory. Due to the small number of cases, multivariate analyses are limited in their ability to determine correlations. The significant association between the GDS and duration of abiraterone acetate use does not confirm a causal effect, although it supports our hypothesis. Nevertheless, longer durations of treatment with abiraterone acetate in our sample of patients were associated with neurocognitive deficits. This correlation was not found for enzalutamide and is the opposite of that found in a disproportionality analysis of a pharmacovigilance database [17]. The neurotoxicity risk was higher with enzalutamide than with abiraterone acetate. However, these authors also note the exploratory nature of their study [17].

One limitation of our study is the nature of our cohort. This incidental sample was evaluated during an outpatient appointment and is relatively small. Moreover, no randomized control group is available. Nevertheless, age- and sex-specific reference scores are available for these neurocognitive tests, which enabled good classification of test results. Our results are important, because studies on cancer- and treatment-related effects in patients with mPC are scarce [18]. As our test battery facilitates cross-comparability of results according to ICCTF guidelines, it can be easily included in future meta-analyses.

Assigning single affected neurocognitive domains to individual treatment-specific characteristics is difficult for several reasons. First, the individual treatment histories of mPC patients are often so different and complex that clear distinctions are not possible. Second, cognitive domains do not function independently, especially in the case of mild impairment. Third, comparability of study results is challenging due to changes in treatment plans and the use of new agents. Fourth, the large number of possible associations between treatments and cognitive domains necessitates very large sample sizes to achieve sufficient statistical power.

In addition to these difficulties, determining the cause of neurocognitive deficits is further complicated by the possible influence of hypogonadism. In patients treated with ADT there are metabolic changes involving the glycemic control and lipid metabolism, increased thrombotic risk, an increased risk of myocardial infarction, severe arrhythmia and sudden cardiac death. Still, these adverse effects can be also due to the subsequent hypogonadism [19]. Therefore, our results should be interpreted only as measures of associations, not as causal relationships.

Nevertheless, in the context of the available evidence, our results indicate that the combination of ADT with a cytostatic or a second-generation antiandrogen is correlated with mild general cognitive impairments. In addition, moderate and severe cognitive impairments were found in a few cases. The identification of single agents responsible for impairment in specific cognitive domains among individuals remains unclear [15].

## Conclusions

- Treatments for mPC significantly are correlated with neurocognitive deficits in several cognitive domains.
- Language skills and processing speed are the most common impairments in mPC patients. However, a consistent pattern of cognitive impairment was not observed.
- Neurocognitive deficits should be considered in phase III and IV trials.

## Data Availability

All data that support the findings of this study are available from the corresponding author upon reasonable request.

## Appendix

See Table 4.

**Table 4:** Multiple regression analyses to predict the GDS criterion by treatment parameter

Predictor	Treatment at the time of testing		Treatment duration/cycles	
	*β*	*p*	*β*	*p*
Drug/surgical castration (LHRH agonist/antagonist or orchiectomy)	− 0.07	*n.s*	− 0.40	< 0.05
Antiandrogen treatment (bicalutamide)	0.10	*n.s*	0.26	*n.s*
CYP17 inhibition (abiraterone acetate)	0.09	*n.s*	0.39	< 0.01
Androgen–receptor inhibition (enzalutamide)	0.18	*n.s*	0.01	*n.s*
Cytostatic drugs	− 0.08	*n.s*	− 0.23	*n.s*
Adjusted *R*^2^ value of the model	*R*^2^ = 0.00	*n.s*	*R*^2^ = 0.17	< *0.05*
